# Complete chloroplast genome sequences of two *Amomum* species (Zingiberaceae)

**DOI:** 10.1080/23802359.2019.1682951

**Published:** 2019-10-26

**Authors:** Ying-Min Zhang, Cong-Wei Yang, Ying-Ying Liu, Yao-Wen Yang, Xiao-Li Liu, Guo-Dong Li

**Affiliations:** a Faculty of Traditional Chinese Pharmacy, Yunnan University of Chinese Medicine, Kunming, China;; b Yunnan Key Laboratory for Dai and Yi Medicines, Yunnan University of Chinese Medicine, Kunming, Yunnan, China;; c Yunnan Institute for Food and Drug, Kunming, China

**Keywords:** *Amomum tsao-ko*, *Amomum paratsaoko*, chloroplast genome, phylogenomic analysis

## Abstract

*Amomum tsao-ko* and *Amomum paratsaoko* are well known medicinal and edible plants with a strong fragrance and flavour in China. Here, we have sequenced the two complete chloroplast genomes of *Amomum tsao-ko* and *Amomum paratsaoko*, which are 163,612 bp and 163,487 bp in length, respectively, and exhibited LSC and SSC regions separated by a pair of inverted repeat regions. The cp genome of *A. tsao-ko* has 120 annotated genes, including 82 protein-coding genes, while *A. paratsaoko* has 121 annotated genes, including 83 protein-coding genes. Both cp genomes contained 30 tRNA genes and 8 rRNA genes. Phylogenetic analysis using a total chloroplast genome DNA sequence of 28 species revealed a close relationship between *A. tsao-ko* and *A. paratsaoko* with 100% bootstrap value.

*Amomum* (Zingiberaceae) are perennial herbaceous plants, and usually endemic to the tropical and subtropical regions, and most of them can be used as pharmaceutical resource (Xian and Yin 2019). The fruits of *Amomum tsaoko* have been used as common materia medica in China, which were considered to treat accumulation of *cold-damp* in the spleen and the stomach with manifestations of epigastric and abdominal distension, fullness sensation and vomiting; and to treat malaria with paroxysms of chills and fever (Chinese Pharmacopoeia Commission 2015). As its commercial value, some similar fruits from the other congeneric species, such as *Amomum paratsaoko*, sometimes are being mixed with *Amomum tsao-ko* as adulterants (Cai et al. 2018). So, it is necessary to develop genomic resources of *Amomum* to provide intragenic information for clarifying the taxonomic identities to ensure the quality of the materia medica, and to illuminate certain questions in the course of evolution of Zingiberaceae.

Two sampled specimens were deposited in the Herbarium of Yunnan University of Chinese Medicine (YNUTCM), China, including *A. tsao-ko* (voucher YYW02-20160806) from the Maguan County, Yunnan Province, China (23°09′N, 104°39′E) and *A. paratsaoko* (voucher YYW15-20160809) from the Baise, Guangxi Province, China (23°10′N, 105°45′E). Total genomic DNA was extracted using plant DNA (Bioteke Corporation, Beijing, China). Genome sequencing was performed on an Illumina HiSeq 2500 platform (Illumina Inc., San Diego, CA, USA). Approximately 6.2 GB clean data were obtained and de novo assembled using NOVOPlasty (Dierckxsens et al. 2017). The complete cp genome was annotated with the online annotation tool GeSeq (Tillich et al. 2017), and annotations were corrected manually with the Geneious R11 11.1.5 (Biomatters Ltd., Auckland, New Zealand).

The circular chloroplast genome of *Amomum tsao-ko* was 163,612 bp (GenBank accession no. MK297333) in size, contained a large single copy (LSC) region of 88,963 bp and a small single copy (SSC) region of 15,355 bp, and separated by a pair of inverted repeat (IRs) regions of 29,646 bp. The cp genome has 120 annotated genes, including 82 protein-coding genes, 30 tRNA genes and 8 rRNA genes. The base compositions of the chloroplast genome were uneven (31.7% A, 18.3% C, 17.7% G, 32.3% T), with an overall GC content of 36.0%.

The complete chloroplast genome of *Amomum para-tsaoko* was 163,487 bp in length (GenBank accession no. MH423780) and composed of IRs of 24,366 bp which divide LSC of 99,398 bp and SSC of 15,357 bp, the average GC content was 36.1% (31.5% A, 18.2% C, 17.9% G, 32.4% T). There are 121 genes annotated, including 83 protein-coding genes, 30 tRNA genes and 8 rRNA genes.

To identify the phylogenetic positions of *A. tsao-ko* and *A. paratsaoko*, 26 representative species from Zingiberales, Liliales, and Dioscroreales were aligned using MAFFT v.7 (Katoh and Standley 2013), and the RAxML (Stamatakis 2014) inference was performed by using GTR model with support for branches evaluated by 1000 bootstrap replicates (Figure 1). Phylogenetic tree supported that two *Amomum* species grouped into one well-supported clade and formed a sister to Zingiberaceae. The complete chloroplast genome of *A. tsao-ko* and *A. paratsaoko* will provide a useful resource for the conservation genetics of this species as well as for the phylogenetic studies of Zingiberaceae.

**Figure 1.**
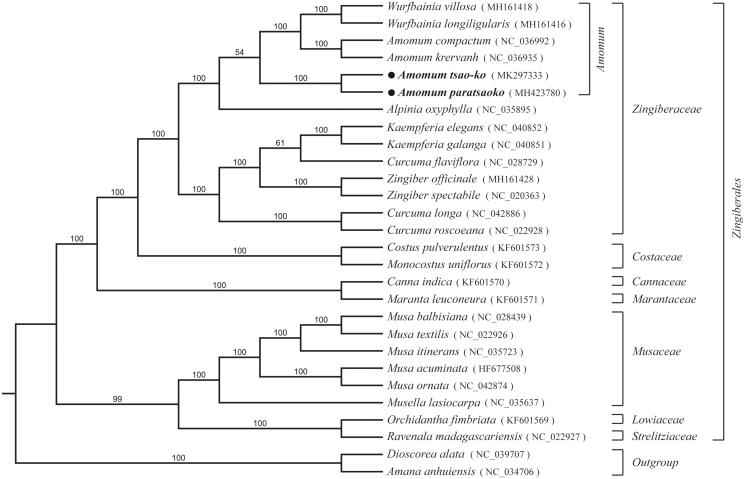
Maximum-likelihood phylogenetic tree inferred from 28 chloroplast genomes. Bootstrap support values >50% are indicated next to the branches.
